# Work addiction among computer engineers : a tunisian study

**DOI:** 10.1192/j.eurpsy.2023.616

**Published:** 2023-07-19

**Authors:** W. Ayed, A. Ayadi, S. Chebbi, S. Ayari, I. Magroun

**Affiliations:** occupational health departement, University of Tunis El Manar - faculty of Medicine of Tunis, Ariana, Tunisia

## Abstract

**Introduction:**

Work addiction is currently an increasingly frequent phenomenon in several sectors of activity, particullarly the engineering sector, given the importance of technological advances and the development of organizational climates favoring competition.

**Objectives:**

To describe work addiction impact among tunisian computer engineers.

**Methods:**

A descriptive cross sectional study was carried out from September first, 2020 to December 31st, 2020 including computer engineers working in several private engineering companies located at Grand Tunis area. Data collection was done through an online self-administered questionnaire. The level of work addiction was assessed by the Work Addiction Risk Test (WRAT) in its French version validated with 25 items evaluating five dimensions namely : Compulsive tendencies, control, lack of communication, inability to delegate and self-esteem. Burnout was identified by The Maslach Burnout Inventory.

**Results:**

A total of 92 computer engineers were included. The average age was 27 ± 4.7 years with extremes ranging from 23 to 55 years. The sex ratio (M / F) was equal to 1.48. Sixty-nine percent (69%) of engineers considered that their work takes so much energy and time that it had a negative impact on their private lives (less time spent with family, more marital conflicts, etc…). Fifty-four percent (54%) of engineers suffered from sleep disorders (difficulty falling asleep, repetitive nocturnal awakenings, etc). The results of the WART questionnaire showed that 58% of engineers were at risk of work addiction, of which 27% had a high risk. This group of engineers with a medium to high risk of work addiction had an average age of 26 ± 3.6 years with extremes ranging from 23 to 42 years. The sex ratio was equal to 1.52. They reported more negative impact of their work on their personal lives (p=0.010) and more reproaches from family members (p= 0.038). They were at risk of burnout syndrome (p=0.038). No statistically significant relationship between the risk of work addiction and the occupational characteristics (occupational category, seniority in the engineering position, number of hours spent at work / week, etc.) was found.

**Conclusions:**

The occupational physician has a crucial role in screening work addiction and its consequences among engineers. Early detection among at-risk populations must be carried out by a multidisciplinary team for appropriate management of workaholic employees.

**Disclosure of Interest:**

None Declared

